# Quality of Life, Stress, and Mental Health in Parents of Children with Parentally Diagnosed Food Allergy Compared to Medically Diagnosed and Healthy Controls

**DOI:** 10.1155/2016/1497375

**Published:** 2016-06-27

**Authors:** Gurkiran Birdi, Richard Cooke, Rebecca Knibb

**Affiliations:** Psychology, School of Life and Health Sciences, Aston University, Birmingham B4 7ET, UK

## Abstract

*Background*. Food allergy is related to poorer quality of life (QoL) and mental health of caregivers. Many parents diagnose food allergy in their child without seeking medical care and there is limited research on this group. This study investigated parental QoL and mental health in parents of children with parent-diagnosed food allergy (PA), medically diagnosed food allergy (MA), and a control group with no allergy (NA).* Methods*. One hundred and fifty parents from a general population completed validated measures of QoL, anxiety, depression, and stress.* Results*. Parents of children with food allergy (PA or MA) reported higher stress, anxiety, and depression than the control group (all *p* < 0.05). Parents of children with MA reported poorer food allergy related QoL compared to parents of children with PA (*p* < 0.05); parents of children with PA reported poorer general QoL compared to parents of children with MA (*p* < 0.05).* Conclusion*. Parents of children with food allergy have significantly poorer mental health compared to healthy controls, irrespective of whether food allergy is medically diagnosed or not. It is important to encourage parents to have their child medically tested for food allergy and to recognise and refer for psychological support where needed.

## 1. Introduction

Food allergy is an immunological reaction to the protein in food which causes rapid symptoms such as swelling and tingling of lips and tongue, rash, vomiting, and in some cases breathing difficulty and anaphylactic shock [1]. Approximately 6–8% of children have a medically diagnosed food allergy [1] and the United Kingdom has one of the highest incidences of allergy in the world [2]. Rona et al. [3] found levels of self-diagnosed food allergy to be as high as 35% although a large proportion of self-diagnosed or parentally diagnosed food allergy cannot be confirmed when participants are brought in for clinical tests such as food challenges [4].

Research on food allergy demonstrates that those caring for children with food allergies report a poorer quality of life for themselves and their children compared to both healthy controls and children with other long-term conditions [5–8]. The unpredictable and potentially life-threatening nature of food allergy in young children has been associated with anxiety and stress in caregivers [7, 9, 10]. Bollinger et al. [11] found that 41% of parents indicated an increase in stress levels since the diagnosis of their child. Activities such as family social events and parties are often affected and many parents try to minimise the anxiety caused by such activities by avoiding them [11, 12]. Research suggests that health professionals are underprepared to address the needs of parents who have children with severe food allergies due to a lack of knowledge concerning this phenomenon [13].

There is little research on the quality of life of parents who have diagnosed food allergy in their child themselves, rather than seeking a medical diagnosis, although it is suggested that these parents may suffer from similar issues. Marklund et al. [14] asked parents of children with parent-reported food hypersensitivity to complete generic quality of life questionnaires and found that parental emotions, time, and familial activities were significantly affected; the younger the child, the greater the impact on family life. By not accessing healthcare for food allergy, families do not have the opportunity to receive guidance and support and it is possible that food allergy could then have a greater impact on quality of life of these families. The aim of this study was to explore the relationship between quality of life, stress, anxiety, and depression in parents of children with parent-diagnosed food allergy and medically diagnosed food allergy and parents of healthy children.

## 2. Methods

### 2.1. Design

This quantitative study was cross-sectional using validated questionnaires measuring quality of life, anxiety, depression, and stress in parents. Ethical approval was gained from the School of Life and Health Sciences Research Ethics Committee at the university where the study took place, prior to commencing data collection and analysis. All participants gave written informed consent before taking part.

### 2.2. Participants and Procedure

All participants were parents, with at least one child aged 0–11 years, recruited from either a local nursery or local primary school. Questionnaires were also provided online and further parents were recruited through social media such as Facebook and Twitter. After completing written or online consent, parents completed a demographic questionnaire and validated psychometric scales to measure general quality of life (WHOQOL-BREF), stress (PSS), anxiety, and depression (HADS). All parents were asked if their child experienced symptoms after eating food (which was not due to food poisoning, stomach bug, or viral infection). Those who answered yes were asked to state whether the cause of their child's symptoms had been medically diagnosed, who had made the diagnosis, and the type of test used and the results of the test. If there was no medical diagnosis, the parent was asked if they had made a diagnosis themselves and what that was. These parents were also asked to complete questions on the food and symptoms involved and completed a food allergy specific quality of life scale (FAQL-PB). Parents who stated that their child's food allergy had been diagnosed by a clinician using skin prick tests, blood test, or food challenge were allocated to the medical-diagnosed group in line with criteria used in previously published research [15].

### 2.3. Materials

#### 2.3.1. The Perceived Stress Scale (PSS) [16]

Perceived Stress Scale (PSS) is a measure of the degree to which situations in one's life are appraised as being stressful. Items are designed to determine how unpredictable, uncontrollable, and overloaded respondents perceive their lives. The questions ask about feelings and thoughts during the past month and scores range from 0 to 5 with the highest rating indicative of higher perceived stress. The PSS is a reliable measure with Cronbach's alpha reported as *α* = 0.84 [17].

#### 2.3.2. Hospital Anxiety and Depression Scale (HADS) [18]

Hospital Anxiety and Depression Scale (HADS) is a 14-item scale; 7 relate to anxiety and 7 to depression. Each item has a 4-point rating scale, scored from 0 to 3; total scores range from 0 to 42 with a higher score indicative of higher levels of anxiety or depression. The scale has good internal consistency with alphas of 0.93 for anxiety and 0.90 for depression and the scale has good construct validity [19].

#### 2.3.3. The WHOQoL-BREF (WHOQoL Group, 1998) [20]

The WHOQoL-BREF is a 26-item general QoL scale and measures physical, psychological, social, and environmental aspects of an individual's life over the last two weeks, using a 5-point Likert scale. There are also two one-item questions, which look at overall quality of life and satisfaction with health and have a score range of 1 to 5 on each. A higher score indicates better QoL. Analyses of internal consistency, item-total correlations, discriminant validity, and construct validity carried out via confirmatory factor analysis confirm the excellent psychometric properties of the scale, with an overall *α* = 0.88 [21].

#### 2.3.4. The Food Allergy Quality of Life-Parental Burden (FAQL-PB) [22]

The FAQL-PB is a 17-item scale that uses a 7-point Likert scale ranging from 1 (not troubled) to 7 (extremely troubled). Items concern issues such as going on vacation, social activities, worries, and anxieties over the previous week. A higher total score is indicative of greater burden on the parents. The scale has excellent internal consistency in a UK sample with *α* = 0.85 [23].

#### 2.3.5. Demographic and Food Allergy Questionnaire

All participants were required to complete a demographic questionnaire. Further questions were completed by any parent stating their child had a medical diagnosis or parental diagnosis of food allergy or intolerance; this included foods and symptoms involved, age at onset of the condition, and details of any diagnostic tests undertaken. Questions were based on a questionnaire used in previous published research [24]. Participants were also asked to indicate whether their child had other major illnesses or conditions (e.g., cancer, diabetes).

### 2.4. Data Analyses

Statistical analyses were conducted using SPSS for Windows (version 22, SPSS, Chicago, IL, USA). Tests were carried out to explore distribution of the data and three normality indicators were used: Shapiro-Wilk test, Kurtosis and skewness, and box plots which indicated there were 21 outliers in the data which were removed. Post hoc power calculations with alpha set at 0.05 were computed and showed the study to have 96% power to detect differences with medium effect sizes and 99% power to detect differences with large effect sizes between the no-allergy and allergy groups; 61% power to detect differences with medium effect sizes; and 86% power to detect differences with large effect sizes between the medically diagnosed and self-diagnosed groups.

Differences in measures between the no-allergy and allergy groups were analysed using ANCOVAs in order to control for differences in demographics across groups. Relationships across all variables were analysed using Pearson's correlations for continuous data and chi-squares for categorical data; predictors of quality of life were analysed using multiple regression. All tests carried out were two-tailed and significance levels were set at *p* < 0.05.

## 3. Results

### 3.1. Participant Characteristics

A total of 157 parents with complete data were analysed, after removing outliers. A total of 7 parents stated that their child had only intolerance to milk or egg rather than an allergy; 6 of these had been diagnosed by a doctor, and one had been diagnosed by a homeopath. These were removed from the dataset leaving a total of 150 in the final analysis. Of these, 66 (44%) identified their children as having no food allergy and 84 (56%) parents reported they had at least one child with a food allergy. A total of 25 parents stated they had made this diagnosis themselves (parent-diagnosed group) and 59 parents stated their child had a medical diagnosis (medical diagnosis group).

The majority (*N* = 104, 69.3%) of participants were from White/Caucasian backgrounds, 26 (17.3%) were of Indian or Asian ethnicity; 8 (5.3%) were Black/African and 5 (3.3%) Hispanic/Latino. Most participants were female (*N* = 135, 90.0%); 98 (65.8%) had been educated to university level; 110 (73.4%) were in full- or part-time work; 106 (70.7%) were married. There were no significant differences across the three groups for education level, work, or marital status. There were slightly more participants in the medical diagnosis group who were mothers (96.6%) compared to the parental diagnosis group (80.0%) or the control group (87.9%), *χ* ^2^(2) = 5.97, *p* = 0.05. There were also significantly more White/Caucasian participants in the medical diagnosis group (88.1%) compared to the parental diagnosis group (52.0%) and the control group (59.1%), *χ* ^2^(2) = 16.60, *p* < 0.001. Therefore, all analyses comparing groups controlled for parent status and ethnicity.

Foods and symptoms reported by parents with a parental or medical diagnosis of food allergy in their child can be seen in Table 1. The most commonly reported allergy in the medically diagnosed group was cow's milk compared to peanuts for the parentally diagnosed group. The most commonly reported symptom was skin rash or eczema in both groups. In addition to food allergies, other long-term conditions reported were eczema (*n* = 54), hay fever (*n* = 30), asthma (*n* = 32), atopic dermatitis (*n* = 31), gastrooesophageal reflux disease (*n* = 1), glue ear (*n* = 1), dislocation of the hip (*n* = 1), ADHD (*n* = 1), and OCD (*n* = 1).

### 3.2. Differences in Quality of Life, Stress, Anxiety, and Depression between Food Allergy and Healthy Control Groups

The first set of analyses looked at differences between the healthy control group and the food allergy group, with the parentally and medically diagnosed groups combined. There were no significant differences between parents of children with food allergy and healthy controls for general QoL (overall QoL or subdomain scores) (Table 2). Parents of children with food allergy did report significantly higher stress levels than parents of children without food allergy (*F*(1,146) = 9.99, *p* = 0.002, *η*_*p*_^2^ = .064), significantly higher anxiety levels than parents of children without food allergy (*F*(1,146) = 7.83, *p* = 0.006, and *η*_*p*_^2^ = .051), and significantly higher depression levels (*F*(1,146) = 8.28, *p* = 0.005, and *η*_*p*_^2^ = .054) (Table 2).

### 3.3. Differences in Quality of Life, Stress, Anxiety, and Depression between Medical- and Parental-Diagnosed Food Allergy

When comparing parentally and medically diagnosed groups, parents of children who were medically diagnosed with food allergy experienced poorer food allergy specific quality of life compared to parents whose children were parentally diagnosed *F*(1,80) = 5.17, *p* = 0.026, and *η*_*p*_^2^ = .061 (Table 3). There were no significant differences between the parent-diagnosed and medically diagnosed group for any of the subdomains of general QoL. There was, however, a statistically significant difference for overall general QoL with parents who diagnosed food allergy in their child reporting poorer general QoL than parents of children with a medical diagnosis (*F*(1,80) = 4.27, *p* = 0.042, and *η*_*p*_^2^ = .051). There were no significant differences for stress, depression, or anxiety (Table 3).

### 3.4. Differences in Quality of Life, Stress, Anxiety, and Depression between Medically Diagnosed, Parental-Diagnosed, and Healthy Control Groups

In comparing across all three groups, there were no significant differences between parental-diagnosed, medically diagnosed food allergy, and healthy control groups for overall QoL or any of the subdomains of QoL.

There was a statistically significant difference regarding perceived stress between the three groups (*F*(2,145) = 5.88, *p* = 0.004, and *η*_*p*_^2^ = .075). Post hoc Tukey's test demonstrated significantly higher stress levels in the parental-diagnosed group (M = 29.8, SD = 7.52) compared to the no-allergy group (M = 24.6, SD = 7.09, and *p* = 0.005). There was no significant difference between parental- and medically diagnosed groups (*p* > 0.05).

There was a significant difference between the three groups for anxiety levels (*F*(2,145) = 5.70, *p* = 0.004, and *η*_*p*_^2^ = .073). Post hoc analysis revealed significantly higher anxiety in the parental-diagnosed group (M = 11.96, SD = 4.59) compared to the no-allergy group (M = 8.37, SD = 4.71, and *p* = 0.004). There was also a significant difference between the three groups for depression levels (*F*(2,145) = 4.13, *p* = 0.018, and *η*_*p*_^2^ = .054). Tukey's post hoc test revealed that the medically diagnosed group reported higher levels of depressive symptoms (M = 8.80, SD = 5.31) compared to the no-allergy group (M = 6.42, SD = 4.85, and *p* = 0.027).

### 3.5. Predictors of Quality of Life for Parents of Children with a Parental or Medical Diagnosis

In parents of children with a medical diagnosis of food allergy for their child, poorer overall QoL and health related QoL, physical, psychological, social, and environmental QoL, and greater stress levels were related to poorer food allergy specific QoL (Table 4). Quality of life and mental health predictors were entered into a multiple regression model to explore whether variables could predict FAQL-PB scores. The model explained 38.1% (*R* ^2^ = 0.38; Adj *R* ^2^ = 0.30) of the variance in parental burden scores *F*(7,58) = 4.49, *p* = 0.001; however, only stress significantly predicted FAQL-PB scores (Table 5). For parents who diagnosed food allergy in their child, only health related QoL significantly correlated with food allergy specific QoL (Table 4); the regression model was not significant and no variables significantly predicted FAQL-PB scores.

### 3.6. Quality of Life, Mental Health, and Food Allergy Characteristics in the Parent and Child

Parents of male children with food allergy (*n* = 52) reported poorer food allergy related quality of life (M = 80.40, SD = 22.77) compared to parents of females with food allergy (*n* = 32) (M = 62.28, SD = 27.20) (*F*(1,83) = 8.66, *p* = 0.004, and *η*_*p*_^2^ = .098). Those parents whose children had not been to hospital with a food reaction (*n* = 46) reported higher anxiety levels (M = 11.54, SD = 4.25) compared to parents whose children had been to hospital with a food reaction (*n* = 38) (M = 9.16, SD = 5.03) (*F*(1,83) = 5.11, *p* = 0.027, and *η*_*p*_^2^ = .060) and reported higher levels of depression (M = 10.00, SD = 4.70) compared to parents whose children had visited hospital with a food reaction (M = 7.32, SD = 5.51) (*F*(1,83) = 5.24, *p* = 0.025, and *η*_*p*_^2^ = .061).

Parents of children who had asthma (*n* = 32) reported higher stress levels (M = 30.22, SD = 7.43) compared to those whose child did not have asthma (*n* = 52) (M = 26.88, SD = 6.43) (*F*(1,83) = 4.61, *p* = 0.035, and *η*_*p*_^2^ = .055). There were no significant differences between parents of children with atopic dermatitis, eczema, or hay fever and parents of children without these conditions.

Out of those parents who reported that their child had a food allergy, 36 parents said they had food allergy themselves. There were no significant differences in stress, anxiety, depression, or food allergy specific quality of life (FAQL-PB scores) between parents who had food allergy themselves and those who did not. Parents without food allergy reported better physical QoL (M = 26.52, SD = 4.47) than parents with food allergy (M = 23.94, SD = 6.62) (*t*(57.98) = 2.02, *p* = 0.05) and better psychological QoL (M = 21.44, SD = 3.86) than those with food allergy (M = 19.42, SD = 4.07) (*t*(82) = 2.32, *p* = 0.02). There were no significant differences between those parents with food allergy and those without for environmental QoL and social QoL. A Pearson's Chi-squared test showed that there was no significant association between the type of diagnosis received by the parent (medical versus self-diagnosis) and the type of diagnosis received by the child (*χ* ^2^ = 0.013, *p* > 0.05).

## 4. Discussion

This study found that food allergy in the child is related to poorer mental health of parents compared to parents of children with no food allergy. Poorer mental health was present irrespective of whether the food allergy diagnosis was by a clinician or by the parent and was not due to the parent having food allergy themselves. Parents of children with a food allergy reported higher stress, anxiety, and depression compared to parents of children with no food allergy. This supports previous literature showing that caring for a child with food allergy is associated with poorer mental health [7, 25–27]. Interestingly, parents who diagnosed the condition in their child reported higher levels of stress and anxiety than parents of children with no food allergy and parents whose children were medically diagnosed reported higher depression than parents of children with no food allergy. Some of the stress and anxiety in the parents who had diagnosed allergy themselves may be due to the uncertainty of not having a medical diagnosis or a lack of information from healthcare professionals. Vargas et al. [28] found that parents of children with recently diagnosed food allergy have reduced stress and anxiety levels if they are provided access to materials and sources of information which are accurate and easy to understand. Akeson et al. [27] reported that parents felt greater anxiety and worry if they did not have information about food allergy and how to treat a serious reaction. Higher levels of depression may come from having more knowledge from medical sources about the potential life-threatening nature of food allergy, for which there is currently no cure. The mental health measures in the current study were general and questions were not specific to food allergy and so reasons for the increased levels of stress, anxiety, and depression warrant further investigation.

This study also found differences in the type of QoL affected in parents of children with a medical diagnosis compared to parents who made a diagnosis themselves, which has not before been reported in the literature. Parents of medically diagnosed children reported poorer food allergy specific QoL and parents who diagnosed the condition in their child themselves reported poorer general QoL. These findings are surprising as caregivers whose children have a medical diagnosis would be expected to have more information regarding the food allergy and its management, as well as more support from the healthcare system and appropriate medication. However, it could be that parents who diagnose the condition themselves may perceive their child's allergy as less serious or less of a burden and something they are able to manage, resulting in them reporting much better levels of food allergy specific QoL. This may be one reason why they have not sought a medical diagnosis for their child or felt the need for a prescription for an adrenaline autoinjector.

The finding that parents of children with parental-diagnosed food allergy reported poorer general QoL than the medically diagnosed group needs further exploration. It could be due to better education or socioeconomic status in parents who access healthcare and seek medical support, although in this study there were no significant differences in level of education or work status between the two groups. There were no significant differences in general QoL between the allergy groups and the group of parents whose children had no allergy, a finding which is consistent with past research where parents of children with nut allergy have reported the same or better QoL than the norm scores derived from a healthy UK population [7]. King et al. used a group of medically diagnosed children and parents and so further research is needed on parents who do not seek a medical diagnosis for their child to see if this finding can be replicated.

A number of food allergy and demographic variables were associated with food allergy specific quality of life. Parents of children with asthma reported higher stress levels compared to parents of children without asthma. This result is also in keeping with some previous studies of children with atopic comorbidities [9, 29]. The increased stress may be due to the fear of consequences or to the distress resulting from having to witness episodes of breathlessness [9]. Interestingly, in this study, parents of males reported higher food allergy related parental burden levels than parents of females. Some reasons for gender differences have been highlighted in the literature [30] such as biological vulnerability, risk evaluation, processing of information, and role expectations. Marklund et al. [14] also found that parents of children with food hypersensitivity reported boys as having poorer QoL than girls. The current study adds to this literature, showing that not only is this seen in the patient, but also is reflected in parental burden.

There are a number of limitations to this study. Ninety percent of the participants were female, with significantly more mothers in the group with a medical diagnosis for their child. Research has highlighted gender differences in experiences of parenting a child with food allergy (e.g., [7]) and so we do not know if the relationships found in this study would be seen in fathers. It is difficult to recruit fathers to this type of research and much of the literature in this area reports data from mothers (e.g., [8, 9, 14, 24, 25]). Research that specifically targets fathers is needed. Further, the study sample consisted primarily of females with a Caucasian ethnicity, again particularly in the group with a medical diagnosis for their child; this may be partly due to recruitment techniques such as using an Internet-based method, which may be more accessible and used by Caucasian ethnicities, but the predominance of these characteristics in the medically diagnosed group does reflect the demographic of parent seen in secondary care allergy clinics in the UK. Although this study controlled for parental and ethnic status, a more ethnically diverse participant sample would be useful to explore.

The study had a smaller number in the parental diagnosis groups compared to the other groups, meaning that the study was underpowered to detect differences with small effect sizes. This is due to the difficulty in recruiting this type of participant who does not access healthcare. Recruitment methods such as social media may facilitate recruitment of participants such as this but this only reaches people who are computer literate and have access to social media, which needs to be taken into account. A further limitation with this study was the reliance on parental report of allergy; there was no verification of the medically diagnosed allergy having been received by a healthcare professional. Recruiting all groups from a general population ensured parity across recruitment methods across the three groups and there is no reason for parents to falsely report the type of diagnosis for their child given the anonymous nature of the study; nevertheless, it would be useful to recruit a medical diagnosed group from an allergy clinic in order to ascertain whether self-reporting has affected these results. Finally this study is cross-sectional and cannot make any inferences regarding cause and effect. Longitudinal data for food allergy is almost nonexistent and is needed in order to further delineate causal pathways, particularly for stress and anxiety.

## 5. Conclusions 

This study highlights the significant relationships between food allergy and caregivers' QoL and mental health irrespective of whether they have sought a medical diagnosis for their child or made the diagnosis themselves. Parents who diagnose food allergy in their child should be encouraged to seek a medical diagnosis. There are large discrepancies between self-diagnosed food allergy and medically confirmed food allergy [3] and Knibb and Semper [26] found that 50% of parents taking their child to an allergy clinic for testing had a negative result, so at least some of the parents in the present study may have got the diagnosis wrong and may be restricting food for their child unnecessarily. A medical diagnosis, alongside appropriate information and support from healthcare professionals, may help reduce the burden of food allergy for these parents. Paediatricians should be aware of the psychological impact of food allergy and recognise and refer caregivers for psychological support if needed.

## Figures and Tables

**Table 1:** Symptoms and foods (*n*/%) related to food allergy reported by parents (% is out of the number of foods or symptoms reported by each group).

Foods	Parental diagnosis (*n* = 71)	Medical diagnosis (*n* = 186)	Symptoms	Parental diagnosis (*n* = 152)	Medical diagnosis (*n* = 543)
Peanuts	16 (22.5)	17 (9.1)	Runny/itchy/congested nose	15 (9.9)	79 (14.5)
Other nuts	11 (15.5)	17 (9.1)	Coughing/sneezing	6 (3.9)	28 (5.2)
Cow's milk	12 (16.9)	49 (26.3)	Itchy skin	19 (12.5)	37 (6.8)
Eggs	11 (15.5)	27 (14.5)	Skin rash/eczema/scabby skin	45 (29.6)	108 (19.9)
Fish	4 (5.6)	7 (3.8)	Dry skin	13 (8.6)	35 (6.4)
Shellfish	2 (2.8)	5 (2.7)	Diarrhoea	5 (3.3)	38 (7.0)
Soya/wheat	10 (14.1)	31 (16.7)	Vomiting	6 (3.9)	37 (6.8)
Fruit	2 (2.8)	20 (10.8)	Swelling of face/tongue	4 (2.6)	40 (7.4)
Vegetables	2 (2.8)	10 (5.4)	Bloated stomach	10 (6.6)	33 (6.1)
Sesame	0	3 (1.6)	Abdominal pain	11 (7.2)	42 (7.7)
Chocolate	1 (1.4)	0	Wheezing/breathlessness/ asthma/tight chest	18 (11.8)	66 (12.2)

**Table 2:** Quality of life, anxiety, depression, and stress mean scores (standard deviations) for parents of children with and without food allergy.

	No-allergy group M (SD)	Allergy group^a^ M (SD)
*WHOQoL-BREF*		
Overall QoL	3.76 (.98)	3.75 (.96)
Overall health related QoL	3.59 (.89)	3.65 (.90)
Physical QoL	26.41 (4.28)	25.42 (5.60)
Psychological QoL	21.29 (3.97)	20.57 (4.05)
Social QoL	10.67 (2.35)	10.10 (2.53)
Environmental QoL	28.70 (4.80)	28.21 (5.71)
*Perceived Stress Scale*	24.61 (7.09)^*∗*^	28.15 (6.98)^*∗*^
*HADS anxiety*	8.38 (4.71)^*∗*^	10.46 (4.74)^*∗*^
*HADS depression*	6.42 (4.85)^*∗*^	8.79 (5.23)^*∗*^

^*∗*^ *p* < 0.01.

^a^Medical and parental diagnosis groups combined.

**Table 3:** Quality of life, stress, depression, and anxiety mean scores (standard deviations) for parents of children who had been medically diagnosed with food allergy and parents who diagnosed food allergy in their children.

	Parental diagnosis M (SD)	Medical diagnosis M (SD)
*FAQL-PB*	59.28 (26.09)^*∗*^	79.53 (23.61)^*∗*^
*WHOQoL-BREF*		
Overall QoL	3.36 (1.08)^*∗*^	3.92 (.86)^*∗*^
Overall health related QoL	3.68 (.75)	3.64 (.96)
Physical QoL	26.76 (4.10)	24.85 (6.07)
Psychological QoL	21.36 (3.82)	20.24 (4.14)
Social QoL	10.12 (2.95)	10.08 (2.37)
Environmental QoL	27.80 (5.07)	28.39 (5.99)
*Perceived Stress Scale*	29.80 (7.52)	27.46 (6.68)
*HADS anxiety*	11.96 (4.60)	9.83 (4.70)
*HADS depression*	8.76 (5.13)	8.80 (5.31)

^*∗*^ *p* < 0.05.

**Table 4:** Pearson's correlations between food allergy specific quality of life, stress, and mental health in the parental and medially diagnosed groups.

	Parental diagnosis FAQL-PB	Medical diagnosis FAQL-PB
*WHOQoL-BREF*		
Overall QoL	−.24	−.30^*∗*^
Overall health related QoL	−.44^*∗*^	−.26^*∗*^
Physical QoL	−.12	−.42^*∗∗*^
Psychological QoL	−.16	−.43^*∗∗*^
Social QoL	.01	−.43^*∗∗*^
Environmental QoL	.14	−.42^*∗∗*^
*Perceived Stress Scale*	.07	.55^*∗∗*^
*HADS anxiety*	−.27	−.03
*HADS depression*	−.24	−.04

^*∗*^ *p* < 0.05, ^*∗∗*^ *p* < 0.01.

**Table 5:** Predictors of food allergy specific quality of life in parents who had a medically diagnosis of food allergy for their child.

	Unstandardised *β*	Standardised *β*	95% CI	
			Upper	Lower
Overall QoL	−2.40	−.09	−11.82	7.01
Overall health related QoL	2.34	.10	−5.29	9.96
Physical QoL	−.60	−.15	−2.10	.91
Psychological QoL	−.27	−.05	−2.72	2.18
Social QoL	−1.16	−.12	−4.42	2.09
Environmental QoL	−.13	−.03	−1.56	1.30
Stress	1.47	.42^*∗*^	.48	2.46

^*∗*^ *p* < 0.01.
